# Oviduct Histopathology of Internal Laying and Egg-Bound Syndrome in Laying Hens

**DOI:** 10.3390/vetsci10040260

**Published:** 2023-03-29

**Authors:** Marina Hosotani, Sohei Hamano, Tomohito Iwasaki, Yasuhiro Hasegawa, Hiromi Ueda, Takafumi Watanabe

**Affiliations:** 1 Laboratory of Veterinary Anatomy, School of Veterinary Medicine, Rakuno Gakuen University, Ebetsu 069-8501, Hokkaido, Japan; 2 Department of Food Science and Human Wellness, College of Agriculture, Food and Environment Science, Rakuno Gakuen University, Ebetsu 069-8501, Hokakido, Japan

**Keywords:** cilia, chicken, egg-bound syndrome, histology, internal laying, oviduct, reproductive disorder

## Abstract

**Simple Summary:**

Internal laying and egg-bound syndromes are avian reproductive disorders that reduce egg productivity in laying hens. To date, the understanding of the importance of peristalsis abnormality in the smooth muscle of oviducts has been focused on the pathogeneses of internal laying and egg-bound syndrome, and the histopathology of the oviductal epithelium has not been explored. In this study, we histologically examined the oviductal ciliated epithelium of aged laying hens. We observe that the epithelial region lacking cilia is larger in the oviducts of hens with internal laying and egg-bound syndrome than in those of healthy hens. In addition, the lamina propria of the oviducts of hens with internal laying and egg-bound syndrome is affected by the infiltration of CD3-positive T-cells. The histological alternation of the ciliated epithelial cells in the oviducts owing to the oviductal inflammation is suggested as the underlying cause of the pathogeneses of internal laying and egg-bound syndrome.

**Abstract:**

In the egg industry, common reproductive disorders, such as internal laying and egg-bound syndrome, not only reduce egg productivity but also cause deaths in severe cases. In this study, we focused on the oviduct histology of the pathogenesis of internal laying and egg-bound syndrome. We divided the aged laying hens into four groups according to the observation of the abdominal cavity and oviductal lumen: healthy, internal laying, egg-bound, and intercurrent. The percentages of healthy, internal laying, egg-bound, and intercurrent groups were 55%, 17.5%, 15%, and 12.5%, respectively. In all parts of the oviduct (i.e., infundibulum, magnum, isthmus, and uterus), the oviductal epithelium was composed of ciliated epithelial cells and secretory cells. The epithelial region lacking cilia was larger in the entire oviduct of the internal laying, and intercurrent groups than in the healthy group. In the internal laying, egg-bound, and intercurrent groups, significant T-cell infiltration was observed in the lamina propria of the entire oviduct. The morphological alteration of ciliated epithelial cells in the oviducts caused by inflammation may be the underlying cause of the pathogenesis of internal laying and egg-bound syndrome.

## 1. Introduction

Selective breeding of laying hens has achieved an approximately two-fold higher egg productivity over the decades [1,2]; however, this has led to the emergence of reproductive disorders. Caged layer fatigue owing to the excess demand for calcium for frequent egg laying includes not only osteopenia but also oviductal inertia because muscle contraction requires calcium [3]. Laying hens spontaneously increase the risk of ovarian cancer with aging, along with decreased egg productivity [4,5]. In addition to genetic factors, rearing circumstances, aging, and infectious diseases also affect the reproductive function of laying hens. Among the common reproductive disorders in laying hens, internal laying and egg-bound syndrome are well-known to reduce egg productivity, and are fatal in severe cases [4,6].

Internal laying is a condition generally observed during inspections in poultry slaughters, where yolk and/or eggs escape into the abdominal cavity [4]. The eggs in the abdominal cavity are absorbed by the immune reaction under healthy conditions [7], whereas they lead to peritonitis under abnormal systemic conditions, including bacterial infection, malnutrition, and stress, ultimately resulting in poultry death [4]. The pick-up failure of ovulated yolks caused by the infundibulum of oviducts and retrograde of eggs via abnormal muscular peristalsis in oviducts constitutes a considerable etiology of the internal laying; however, the oviduct histopathology remains unexplored. Egg-bound syndrome is a disease in which one or more eggs stagnate in the oviduct. Fatigued muscular peristalsis in the oviducts owing to hypocalcemia, stress, and aging is involved in egg-bound syndrome; the reduced quality of egg components also alters the oviductal transportation efficiency [4,6]. Although oviductal muscular insights have been hypothesized in previous studies, there are no reports focusing on oviduct histopathology related to egg-bound syndrome.

The avian oviducts composed of ciliated epithelium, lamina propria distributing tubular glands, and smooth muscle layer govern egg maturation and transportation, including yolk pick-up, fertilization, and egg formation [8,9]. The infundibulum, magnum, isthmus, uterus, and vagina, in order from the cranial to caudal parts, are divided and regulate the dependent physiological functions [10]. Except for the distribution of tubular glands playing roles in egg formation, avian oviductal histology replicates the mammalian one; therefore, the histopathological changes related to oocyte transportation in mammalian oviducts are hypothesized to be closely related to the pathogenesis of the internal laying and egg-bound syndrome in laying hens. Indeed, blockage of smooth muscle activity in murine oviducts impairs oocyte transportation [11]. In mammals, alterations in the oviductal ciliated epithelium trigger oocyte pick-up and transportation dysfunction. The loss of ciliated epithelial cells, randomized ciliary orientations, and changed ciliary beating speed have been reported to cause dysfunction in oocyte pick-up and transportation [12,13,14]. Therefore, in this study, we explored the histopathology of the oviductal ciliated epithelium and aimed to test the hypothesis that the abnormality of ciliated epithelium is closely related to the incidence of internal laying and egg-bound syndrome in laying hens.

## 2. Materials and Methods

### 2.1. Animals and Sample Collection

Animal experiments were approved by the Institutional Animal Care and Use Committee of Rakuno Gakuen University (No. VH20A17). Eighty Japanese laying hens (*Sakura* as the hybrid strain of White Leghorn, Rhode Island Red, and White Plymouth Rock and *Momiji* as the hybrid strain of Rhode Island Red and White Plymouth Rock) ranged from 500 days to 850 days old were used. These hens were in the group that was subjected to be discarded due to overall aging and declining production rates. As chickens show a 25 h egg-laying cycle and lay eggs in the morning, all the samplings were conducted in the afternoon. All hens were euthanized via cardiac blood collection and cutting carotid artery under anesthesia induced by pentobarbital sodium (80 mg/kg). All hens were divided into four groups according to observations of the abdominal cavity and oviductal lumen: “internal laying” as individuals with eggs in the abdominal cavity; “egg bound” as those with one egg in the infundibulum, magnum, or isthmus, or those with two or more eggs in the oviductal lumen; “intercurrent” as those with both aforementioned findings; and “healthy” as those without the aforementioned findings. The infundibulum, magnum, isthmus, and uterus of the oviducts were collected, fixed with 4% paraformaldehyde overnight at 4 °C, and embedded in paraffin.

### 2.2. Measurement of Serum Calcium Level

Serum calcium level was measured with a Metallo assay LS kit for calcium (CPZ III, MG Metallogenics, Chiba, Japan) according to the manufacturer’s instructions.

### 2.3. Histology

The embedded tissues were sliced into 3 µm thick histological sections, which were subjected to hematoxylin and eosin (HE) staining, periodic acid–Schiff (PAS) staining, and immunohistochemistry (IHC). For IHC, deparaffinized sections were incubated for 15 min at 110 °C in 20 mM Tris–HCl (pH 9.0) (for anti-CD3 antibody). The sections were then soaked in methanol containing 0.3% hydrogen peroxide. After washing thrice in 0.01 M phosphate-buffered saline (PBS), the sections were incubated with 10% normal rabbit blocking serum for 60 min at room temperature, and then incubated overnight at 4 °C with anti-α-tubulin antibody (ready to use; MS-581-R7, ThermoFisher Scientific, Waltham, MA, USA) or anti-CD3 antibody (1:400; F7.2.38, Abcam Inc., Cambridge, UK). Negative controls were performed with normal mouse IgG (sc-2025, Santa Cruz Biotechnology Inc., Santa Cruz, CA, USA). After washing thrice in 0.01 M PBS, the sections were incubated for 30 min with biotin-labeled rabbit anti-mouse IgG + IgA + IgM antibody (ready to use; 426031, Nichirei, Tokyo, Japan). After washing thrice in 0.01 M PBS, the sections were incubated for 30 min at room temperature using a streptavidin–biotin complex (SABPRO Kit, Nichirei), incubated with a 3,3′-diaminobenzidine tetrahydrochloride-hydrogen peroxide solution, and lightly stained with hematoxylin. A Primostar 3 microscope (ZEISS Inc., Oberkochen, Germany) was used to examine stained sections.

### 2.4. Histoplanimetry

Four hens for each group were randomly selected for statistical histoplanimetry. The percentage of α-tubulin-negative region on the epithelium was quantified in five random fields obtained from the oviductal sections stained with IHC for detection of α-tubulin as follows: percentage of α-tubulin-negative region on the epithelium (%) = 100 × the length of the α-tubulin-negative region on the epithelium (µm)/the overall length of the ciliated epithelium in the field (µm). The PAS-positive region in the epithelium was quantified in five random fields obtained from the oviductal sections stained with PAS as follows: PAS-positive region in the epithelium (µm^2^) = the area of the PAS-positive region in the epithelium (µm^2^)/1 mm (as a unit length of the epithelium). The percentage of infiltration region of CD3-positive T-cells in the epithelium was quantified in five random fields obtained from the oviductal sections stained with IHC for detection of CD3 as follows: percentage of infiltration region of CD3-positive T cells in the epithelium (%) = 100 × the area of the CD3-positive regions in the epithelium (µm^2^)/the overall area of the oviduct in the field (µm^2^).

### 2.5. Statistical Analysis

The results are expressed as the mean + standard error (SE). Data from three or more groups were compared using Tukey–Kramer test (*p* < 0.05).

## 3. Results

### 3.1. Gross Pathology and Incident Rate of Internal Laying and Egg-Bound Syndrome

**Figure 1:**
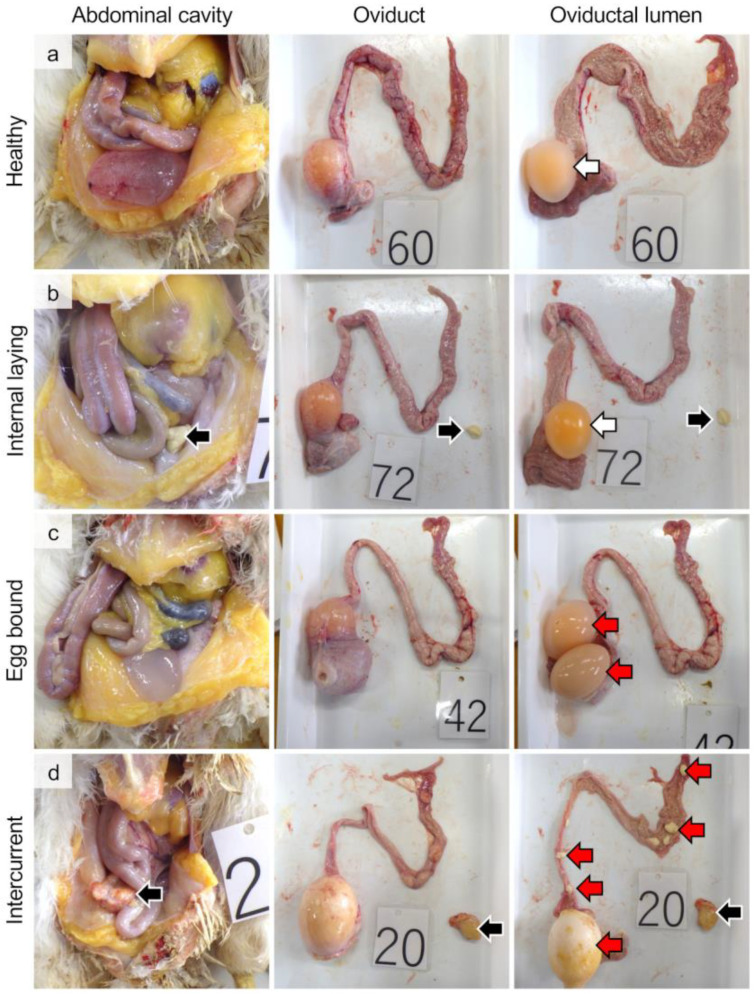
Gross images of abdominal cavity, oviducts, and oviductal lumen of laying hens divided into the groups of healthy (**a**), internal laying (**b**), egg-bound (**c**), and intercurrent (**d**). White arrows: one egg in the uterus. Black arrows: eggs escaped into the abdominal cavity. Red arrows: two or more eggs stagnating into the oviduct.

None of the examined hens showed any clinical symptoms. In the healthy group, no egg components (i.e., yolk, egg albumin, or shells) were found in the abdominal cavity or oviductal lumen (Figure 1a). Laying hens holding only one egg in the uterus were regarded as healthy hens owing to the 25 h laying cycle [15]. In the internal laying group, egg components were observed in the abdominal cavity (Figure 1b). In the egg-bound group, two or more egg components were observed in the oviductal lumen (Figure 1c). In the intercurrent group, more than two egg components were observed in both the abdominal and oviductal lumens (Figure 1d). According to these gross observations, the percentages of the healthy, internal laying, egg-bound, and intercurrent groups were 55%, 17.5%, 15%, and 12.5%, respectively (i.e., n = 44, 14, 12, and 10 per group, respectively).

**Figure 2:**
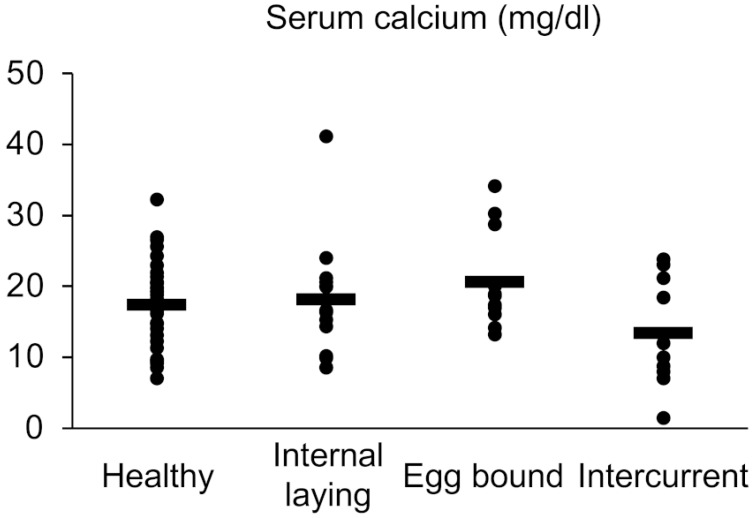
Serum calcium level in laying hens. There are no significant differences among groups (Tukey–Kramer test, n = 44, 14, 12, 10 for groups of healthy, internal laying, egg-bound, and intercurrent, respectively). The averages for each group are presented as the bars.

To reveal the metabolic conditions related to eggshell production and oviductal muscular peristalsis, we analyzed the serum calcium level in the laying hens; however, there were no significant differences among the groups (Figure 2).

### 3.2. Oviduct Histopathology Focusing on the Ciliated Epithelium

**Figure 3:**
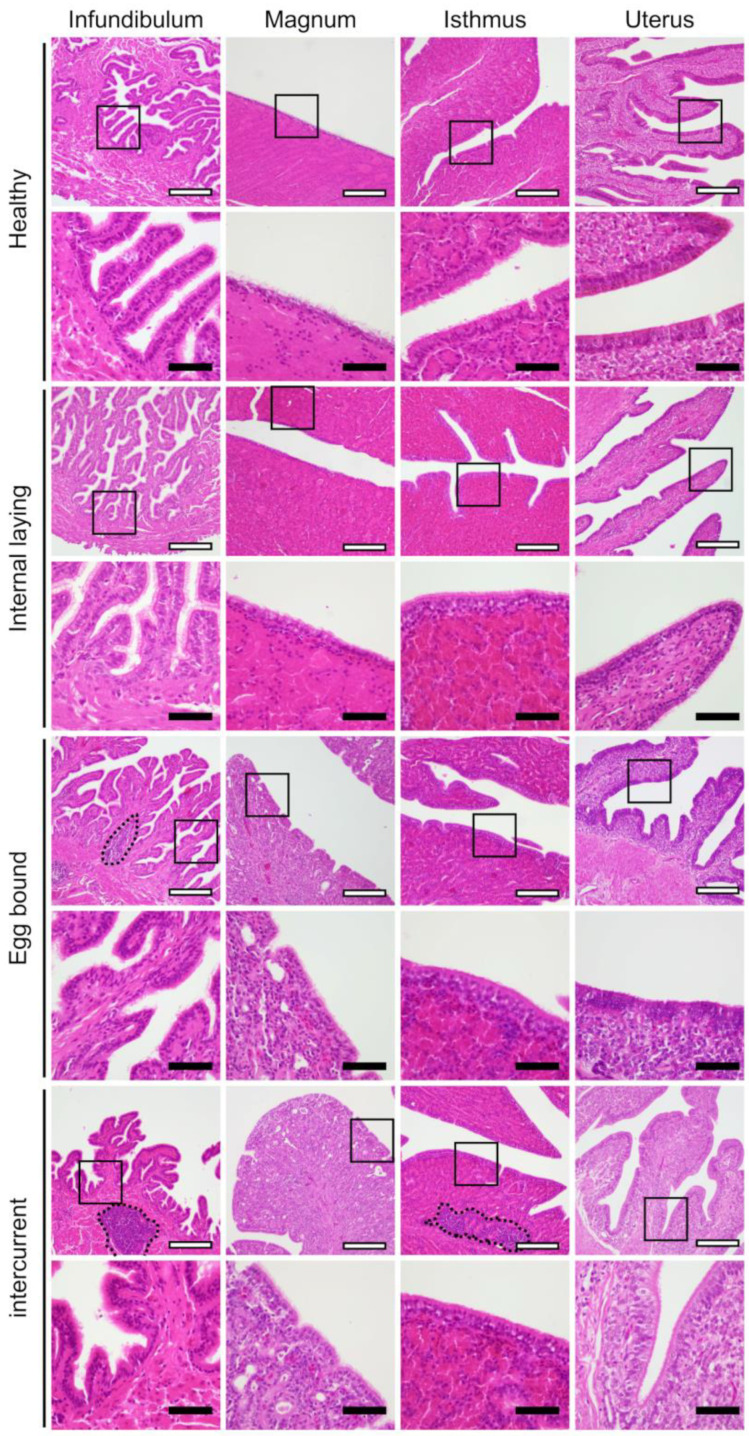
Histology of infundibulum, magnum, isthmus, and uterus of oviducts in laying hens. The squared regions are magnified into the below images. Dotted lines indicate the infiltration of mononuclear cells. White bar = 200 µm, black bar = 50 µm.

There are no noteworthy differences in the histology of the smooth muscle layer among the groups. Mononuclear cell infiltration was observed in the oviductal lamina propria in the affected groups (i.e., the groups of internal laying, egg-bound, and intercurrent), but not in the healthy group (Figure 3).

**Figure 4:**
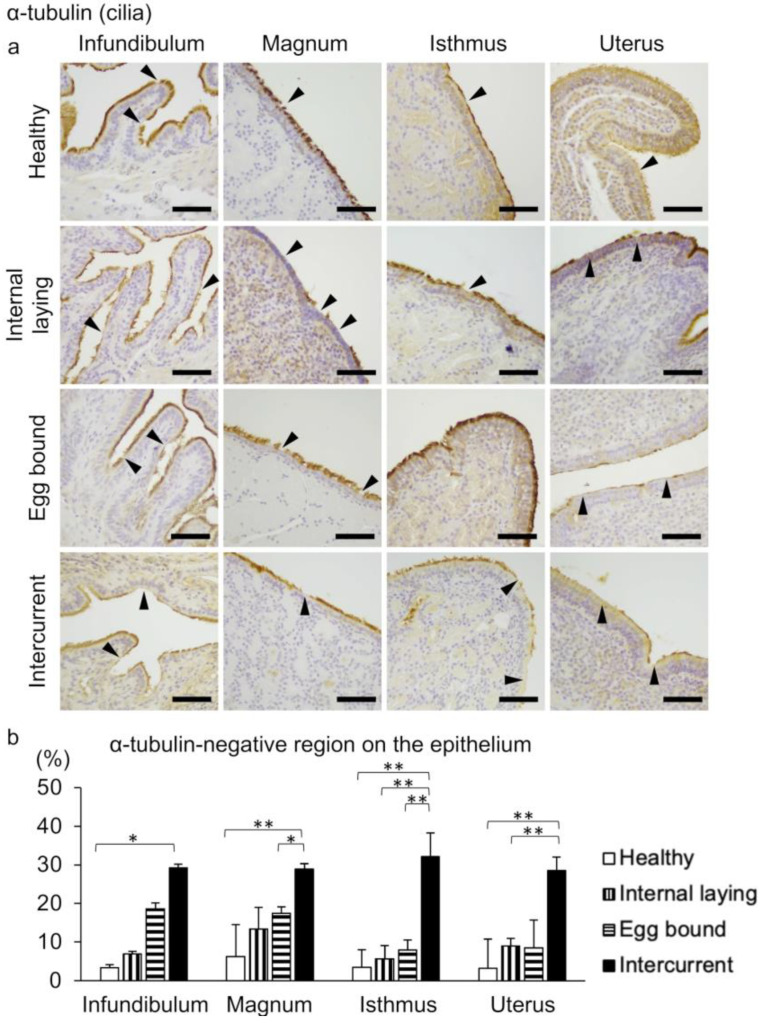
Deficiency of cilia on the oviductal epithelium in laying hens. (**a**) Immunohistochemistry for detecting α-tubulin-positive cilia on the ciliated epithelial cells of oviducts. Arrowheads indicate the α-tubulin-negative regions on the epithelium. Bar = 50 µm. (**b**) The percentage of α-tubulin-negative region on the oviductal epithelium. * *p* < 0.05, ** *p* < 0.01 (Tukey–Kramer test, n = 4 for each group). Data are presented as the mean + SE.

**Figure 5:**
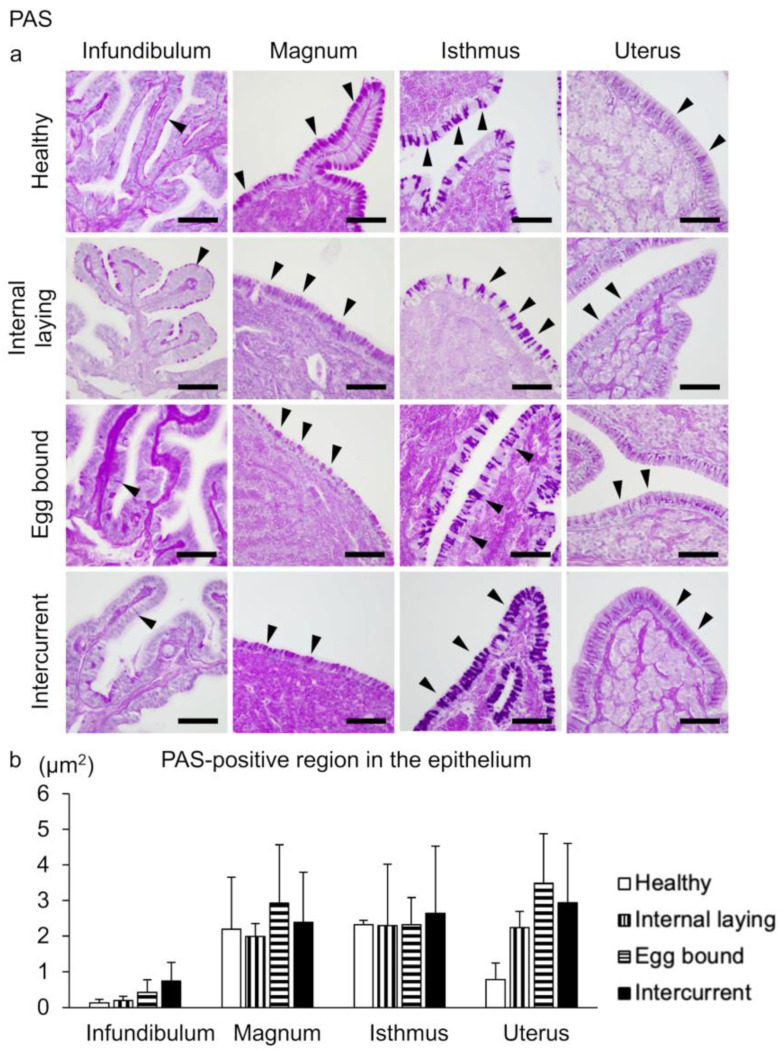
Secretory cells distribution in the oviductal epithelium in laying hens. (**a**) Periodic acid–Schiff stain (PAS) for detecting secretory cells of oviducts. Arrowheads indicate the PAS-positive secretory cells on the epithelium. Bar = 50 µm. (**b**) The percentage of PAS-positive area in the epithelium. There are no significant differences among groups (Tukey–Kramer test, n = 4 for each group). Data are presented as the mean + SE.

The avian oviductal epithelium consists of ciliated epithelial cells and secretory cells. The former cells were visualized by detecting cilia through IHC using an anti-α-tubulin antibody (Figure 4), and the latter cells were visualized using PAS (Figure 5). The negative control of IHC is presented in Supplementary Figure S1. The α-tubulin-negative regions on the epithelium are observed in the entire oviduct; however, the regions tend to be larger in the affected groups than in the healthy group (Figure 4a). Histoplanimetric analysis reveals significantly larger α-tubulin-negative regions in the epithelium of the intercurrent group than in the healthy group (Figure 4b). The distribution of PAS-positive secretory cells is observed in the entire oviduct of all hens, with no significant differences among the groups (Figure 5a,b).

### 3.3. Inflammation in the Oviducts of the Laying Hens with Internal Laying and Egg-Bound Syndrome

**Figure 6:**
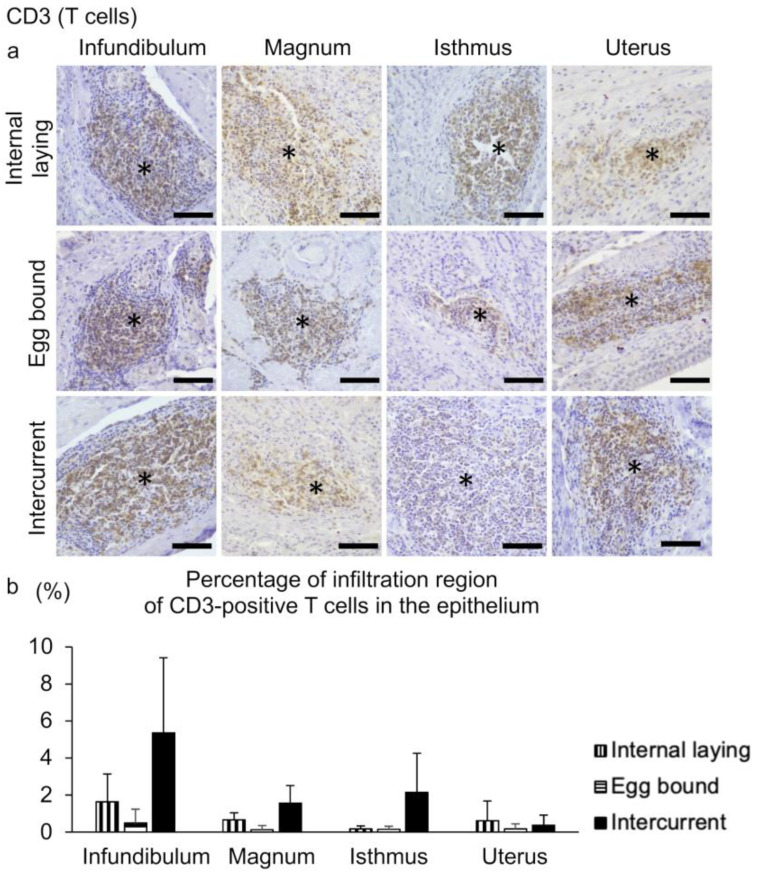
Infiltration of CD3-positive T-cells in the lamina propria of oviducts in the group of internal laying, egg-bound, and intercurrent. (**a**) Immunohistochemistry for detecting CD3-positive T-cells in the lamina propria of oviducts. (*) Asterisks indicate the center of lymphoma-like infiltration of CD3-positive T-cells. Bar = 50 µm. (**b**) The percentage of infiltration region of CD3-positive T-cells in the epithelium. There are no significant differences among groups (Tukey–Kramer test, n = 4 for each group). Data are presented as the mean + SE.

To identify mononuclear cells infiltrating the lamina propria of the affected groups, IHC using anti-CD3 antibody as a T-cell marker was performed. Prominent infiltration of CD3-positive T cells is observed in the lamina propria of the entire oviduct in the affected groups (Figure 6a). Mononuclear cell infiltration is not observed in the healthy group, so that we do not present the IHC in the healthy group. Histoplanimetric analysis reveals that the percentage of infiltration region of CD3-positive T-cells in the epithelium shows no significant differences among the affected groups (Figure 6b).

## 4. Discussion

Forty-five percent of the laying hens we examined show internal laying and/or egg-bound syndrome, indicating that aged laying hens are highly affected by reproductive disorders. On the other hand, none of the examined hens show severe clinical findings, such as body weight loss and change in respiration rate. These results suggest that severe cases of internal laying and/or egg-bound syndrome that threaten life are not frequent in aged laying hens. Egg productivity and quality deteriorate as hens grow older [8,16], along with systemic metabolic and endocrine alterations in the kidneys and liver [17,18]. Therefore, changes in the systemic and/or oviductal metabolic and endocrine profiles affected by aging are thought to be the underlying causes of the pathogeneses of internal laying and egg-bound syndrome.

In this study, we observe no histological changes in the oviductal muscle layer and no significant differences in serum calcium levels among the groups as the energy source of muscular peristalsis in the affected groups compared to the healthy group. However, we observe the eggs with shell membrane in the abdominal cavity (Supplementary Figure S2). A previous study also reports that ovulated yolk, egg albumen, and eggs with shells are sometimes found in the abdominal cavity of laying hens [19]. Abnormal egg morphology, such as large eggs, also disturbs smooth egg transportation [6]; however, there are no significant differences in the histology of tubular glands between the healthy and affected groups. Therefore, it is suggested that the eggs retrograde from the uterus to the abdominal opening in the infundibulum owing to abnormal oviductal muscular peristalsis, oviductal inertia, and external pressure on the oviducts, which stagnate into the oviducts or escape into the abdominal cavity. In addition, peristalsis in the muscle layer of the magnum regulates yolk pick-up by closing the infundibulum to the ovary at ovulation [20], implying that muscular dysfunction in the cranial oviducts may cause pick-up failure and internal laying. Further functional analysis of the muscle layer of the oviducts would emphasize its importance in the pathogeneses of internal laying and egg-bound syndrome.

Importantly, we found a tendency of fewer cilia-covering regions on the oviductal epithelium in the groups with internal laying and egg-bound syndrome compared to the healthy group, and significant ciliary defects in the intercurrent group. In mammals, the decreased number of ciliated epithelial cells in the infundibulum causes oocyte pick-up disorder [12]; the complete defect of ciliated epithelial cells in the entire oviduct reduces the oocyte transportation efficiency [14]. Therefore, the altered distribution of cilia is thought to be closely associated with internal laying and egg-bound syndrome. In contrast, there are no significant differences in the distribution of secretory cells among the groups. In mice, oviductal epithelial homeostasis is maintained via self-proliferation and differentiation of secretory cells into ciliated epithelial cells [21]. Although the cell type of the epithelial progenitor in laying hens has not been verified [22], our results imply that the differentiation of epithelial progenitor cells into ciliated epithelial cells is disturbed, leading to an increase in the number of the abnormal ciliated epithelial cells lacking cilia.

The infiltration of T-cells in the oviductal lamina propria is a distinctive characteristic of the affected groups compared to the healthy group, suggesting that inflammation in the oviduct is the key factor leading to internal laying and egg-bound syndrome. In mice, significant inflammation caused by CD3-positive T-cells disturbs oviductal epithelial homeostasis, resulting in a decreased number of ciliated epithelial cells and uncoordinated ciliary orientation [12,23,24]. In addition, inflammatory cytokines, including IL-4, IL-5, IL-6, and IL-13, produced by T-cells, decreased the ciliary beating ability in mice and humans [25,26]. Furthermore, salpingitis triggers the production of prostaglandins, causing reverse muscular peristalsis in the oviducts of laying hens [4]. Salpingitis mediated by T-cells interacts closely with the abnormal morphofunction of the ciliated epithelium and muscular functions of oviducts; therefore, in laying hens, inflammation is the fundamental pathological process of internal laying and egg-bound syndrome. We did not determine the principal factors of oviductal inflammation in this study, although salpingitis in laying hens is reported to be caused by aging [27] and general bacterial infections, such as those caused by *Escherichia coli* and *Salmonella* [6,19,28,29]. The laying hens infected with infectious bronchitis virus during chick-hood exhibit oviduct atrophy, resulting in a significant reduction in egg production and a high appearance of internal laying [30]. Therefore, appropriate rearing circumstances considering relief from stress and infection could suppress the incidence of internal laying and egg-bound syndrome.

## 5. Conclusions

Our study provides the histopathological evidence that the oviductal ciliary defects relating to T-cell inflammation underlie the pathogenesis of internal laying and egg-bound syndrome in the aged laying hens. These findings are crucial for reproductive disorders in the avian species.

## Data Availability

The raw data supporting the conclusions of this article will be made available by the authors without undue reservation to any qualified researcher.

## Supplementary Materials

The following supporting information can be downloaded at: https://www.mdpi.com/article/10.3390/vetsci10040260/s1, Figure S1: Negative control of immunohistochemistry using normal mouse IgG. Bar = 50 µm. Figure S2: Gross image of abdominal cavity of hens with internal laying of the eggs with shell membrane (black arrow).
