# Supplementary material for: Oviduct Histopathology of Internal Laying and Egg-Bound Syndrome in Laying Hens
Source: Vet Sci. 2023 Mar 29;10(4):260. doi: 10.3390/vetsci10040260 (PMC10142957; doi:10.3390/vetsci10040260)
Supplement: Supplementary file 1 [file vetsci-10-00260-s001.zip › Figure S1.pdf]

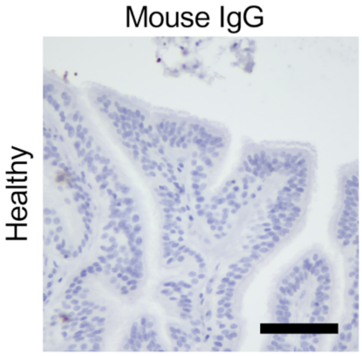

Supplementary Figure S1: Negative controls of immunohistochemistry using normal mouse IgG. Bar = 50 μm.
